# A personal and professional journey - experiences of being trained online to be a supervisor in professional supervision in nursing

**DOI:** 10.1080/17482631.2021.1952523

**Published:** 2021-07-13

**Authors:** Jenny Molin, Monica Öberg-Nordin, Barbro Arvidsson, Britt-Marie Lindgren

**Affiliations:** a Department of Nursing, Umeå University, Umeå, Sweden; b Department of Clinical Sciences, Division of Psychiatry, Umeå University, Umeå, Sweden; c School of Health and Welfare, Department of Health and Nursing, Halmstad University, Halmstad, Sweden

**Keywords:** Education, experiences, leadership, longitudinal, online, process-oriented group supervision in nursing

## Abstract

**Background:**

Nurses often work alone in complex environments with ambiguous responsibilities and need ensured access to supervision.Online supervision has become common and has potential to support supervision in rural areas.

**Aim:**

To explore the experiences of registered nurses (RNs) learning online to be a supervisor in professional supervision in nursing.

**Design:**

A longitudinal qualitative design was used.

**Methods:**

A total of six focus group discussions, with 15 RNs divided in two groups, were conducted before, during, and after the training. Data underwent qualitative content analysis.

**Results:**

Results showed that the participants experienced learning to be a supervisor online as a personal and professional journey, and learning online was an advantage rather than a disadvantage. Initially, they focused on themselves, then on themselves within the group, and finally on themselves and the group. Both the group and the internet environment were described as safe places. Online tutoring needs to include the creation of a social presence within the group.

## Background

Professional supervision is a recognized process of professional support and learning that allows health care staff and students to develop professional competence through guided questions and self- reflection (Edwards et al., 2005). The term is used in a variety of ways with different ideas, approaches and methods. Further, it is synonymous with terms such as “clinical supervison” and “guided supervision” (Martin et al., 2014).

Internationally and in Sweden, training to become a supervisor in professional supervision in nursing is usually conducted according to nationally determined guidelines in a campus environment, with physical meetings for group supervision (Swedish Nursing Association & National Association for Professional Counseling in Nursing, 2008). Professional Supervison in Nursing (YHIO; Swedish abbreviation) is a well-known professional group supervision model in the Nordic countries (Lindgren et al., 2005), influenced by confluent education theory (Franke & Erkins, 1994) and gestalt therapy (Perls, 1977). YHIO emphasizes the combination of affective and cognitive features in the learning process, as well as the impact of both the learning environment and the group leader (Rodgers, 1965) and the reflective process (Gibbs, 1988). The model, used both clinically and for nursing students during their training to become registered nurses, is based on a group contract including structural factors such as voluntariness, confidentiality, continuity, responsibility, and willingness to self-development, and climate factors of genuineness, acceptance, support, trust, empathy, and challenge (Lindgren et al., 2005). A prerequisite of these group sessions has always been that they are conducted face-to-face, with all members in the same place at the same time. Through literature search, no scientific literature was found that describes online forms of professional supervision.

Previous research showed that after a year of taking part in group supervision, supervisors’ improved views of their own abilities and satisfaction in their role increased their ability to supervise students and promoted their own personal development (Borch et al., 2013). The same authors reported that group supervision can be a valuable support to clinical supervisors in bachelor nursing education. Studies on the effects of YHIO on professional nurses unanimously show that supervision in nursing strengthens nurses’ professional development (Francke & de Graaff, 2012). There is research evidence to suggest that supervision provides nurses with peer support and stress relief, promotes their professional accountability, and helps to develop their skills and knowledge (Brunero & Stein-Parbury, 2008; Edwards et al., 2006). Ohlsson and Arvidsson (2005) also show that nurses feel that YHIO helps relieve work stress. All of the above studies on YHIO took place in a campus environment.

In a literature review (2015), Deane and co-workers reported that electronically supported supervision (e.g., telephone) has long been available, is reliable, and has become relatively inexpensive. Supervision via teleconferencing and videoconferencing represent solutions to a particular set of needs: convenience, availability, and overcoming physical distance. Such solutions are used when traditional face-to-face meetings are not convenient or practical (Deane et al., 2015). A literature review suggests barriers to distance education can be categorized as technical, psychological, pedagogical, social, cultural, and contextual (Berge, 2013). To some degree, most of these barriers overlap and merge (Dabaj, 2011).

Supervision conducted online has become more common (Deane et al., 2015), and as it has the potential to support distant supervision in rural areas, it is important to train supervisors in performing supervision online. According to the Community of Inquiry (CoI) model of Garrison et al. (2000), learning in online environments is assumed to occur through the interaction of three core elements: social presence, cognitive presence, and teaching presence. These elements work together to support deep and meaningful online inquiry and learning (Garrison et al., 2001). In our literature search and contacts with national networks and researchers in the field, we found no research on conducting online tutoring for supervisors of YHIO.

## Aim

The aim of this longitudinal study was to explore the experiences of registered nurses (RNs) learning online to be a supervisor in professional supervision in nursing.

## Method

### Research design

This study used a longitudinal inductive qualitative design with semi-structured focus group discussions before, during, and after participating RNs completed the training. Data were subjected to qualitative content analysis (Lindgren et al., 2020; Graneheim et al., 2017; Graneheim & Lundman, 2004).

### Context

In Sweden, to learn how to be a group leader in YHIO, RNs with a least 12 month work experiences, need to take an advanced course namned “Leadership and Professional Supervision in Nursing”, 30.0 credits.

#### Description of the advanced course “leadership and professional supervision in nursing”, 30.0 credits

**Figure 1.**
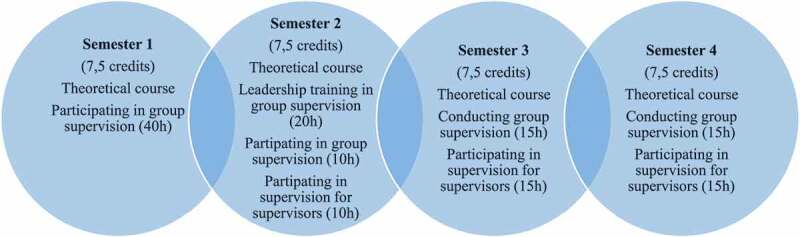
Overview of education

The educational approach in the course Leadership and Professional Supervision in Nursing uses a blended learning design. Blended learning refers to a mixture of different learning environments and combines traditional classroom methods with more modern computer-mediated activities where the innovative challenge was to conduct skills training in online tutoring (Osguthorpe & Graham, 2003). Figure 1 shows an overview of the education.

The course runs part-time at 25% for four semesters with predominantly online gatherings, using a learning management system (LMS), an open source system supporting technology with enhanced teaching, learning, and collaboration available to all employees and students at the university. A web conference solution provided free of charge by the Swedish University Computer Network (SUNET), was used for online conversations. The web conference solution enabled collaboration with live sound and video, document and desktop sharing, and the ability to record meetings. The RNs who took the course connected to the meeting using their own personal computers, web cameras, and internet connections. The course applied a student–active working method including reflecting together with classmates and lecturers. The working methods varied between individual work and group work. One physical class was arranged at the university each semester.

The theoretical content of the course covered the nursing profession; nursing supervision; nursing value base, ethics, and methods; communication; group processes; and leadership, focusing on nursing supervision. The practical training for the RNs included participation in a supervision group (Semester 1) and practice in supervising groups (semesters 2–4). The composition of the groups remained the same during the whole course. The purpose of the training was to develop and reflect upon personal leadership in supervision, planning, and evaluation, based on the group members’ individual needs and conditions and the different phases of the supervision.

### Participants

Sixteen RNs from all over Sweden started the course and all were asked to participate in the study. Fifteen RNs (14 women and 1 man, aged 28–59 years) chose to participate (2 radiographers, 4 in psychiatric care, 2 in elderly care, 3 in primary health care, 1 in palliative care, 2 in internal medicine, 1 in cardiology). Seven of them worked as lecturers in nursing during the course.

### Data collection

Focus group discussions were chosen for data collection for their ability to elicit qualitative data from the interaction between group members in enhanced discussions (Peek & Fothergill, 2009).

The participants were divided into two focus groups. To facilitate cohesion these were the same groups that had supervision together during the course. Group 1 consisted of seven participants and Group 2 of eight.

Due to the longitudinal design, the focus group discussions were conducted on three occasions during the two years of training. Data collection took place between September 2014 and May 2016. The first occasion was conducted during the first week of the course and focused on nurses’ experiences of taking the course and their thoughts about supervisors and supervision. The main question was; *Can you tell me about your thoughts on starting this course and on leadership?* The second occasion was conducted after one year of training and the discussion focused on what it means to be a supervisor and the participants’ experiences of online supervision. The main question was; *Tell me*, w*hat does it mean to be a supervisor and a leader?* The third occasion was conducted after the course finished and focused on the participants’ experiences of the training as a whole. The main questions were; *Can you tell me how it was to take this course online? How was it to supervise own groups?*

The focus group discussions took place in a room at the university and lasted from 52 to 86 minutes (median 62 minutes). At the second focus group discussion, three participants were unable to participate, and at the third, four participants were absent. The third author (BA), an RN with advanced training in supervision and use of scientific methods in nursing, moderated and kept notes of the discussions. The focus group discussions were audio-taped and transcribed verbatim.

### Data analysis

The analytic method chosen was qualitative content analysis (Lindgren et al., 2020; Graneheim et al., 2017; Graneheim & Lundman, 2004). First, the texts were read as a whole to get a sense of the nurses’ overall experiences of the course. Then the texts were divided into meaning units, which were condensed (shortened without losing any content), abstracted, and each given a code. The codes were then sorted, based on similarities and differences, abstracted, and interpreted into subthemes. For example, codes such as “new challenges”, “moving on”, “development”, and “possibilty to advance” were grouped, abstracted, and interpreted into the subtheme of “expectations”. Subthemes with similar content were grouped together, abstracted, and interpreted to formulate broader themes and the main theme. Three of the authors (JM, MÖ-N, B-ML) conducted the analysis and discussed the results until they reached consensus. JM and MÖN had previous knowledge on the course while BML did not. Assumptions and self- reflections made by the researchers were highlighted and discussed. Further, interpretations were controlled by moving back to data.

### Ethical considerations

The project was carried out in accordance with the Declaration of Helsinki (World Medical Association, 2013). According to Swedish legislation, healthy adult people are not considered vulnerable participants in research and therefore ethical approval is not required. Instead, approval of the study was obtained from the head of the department at the University. All RNs participated voluntarily after being given verbal and written information and all gave their written informed consent. They were informed of their ability to withdraw at any time without stating any reason and without jeopardizing their education.

## Results

The results showed that learning to be a supervisor in professional supervision in nursing online was a personal and professional journey and that learning online was an advantage rather than a disadvantage. The results are presented in a linear way; however, the participants’ learning processes went back and forth throughout the whole course. Table I shows an overview of the subthemes and themes revealed in the analysis.

**Table I.** Overview of the results

Subtheme	Theme	Main theme
Facing fear Having expectations	Zooming in on oneself	
Having ideals of the role Using the group as a mean	Focusing on oneself in the group	Going on a personal and professional journey
Feeling strengthened Travelling with others Having a safe place	Shifting perspectives on oneself and the group	

### Going on a personal and professional journey

The nurses’ learning is described as a process in which the participants initially focused on themselves. Along the way, they began to focus on themselves within the group. During the last semester, the participants shifted their perspectives on themselves and on the group.

#### Zooming in on oneself

The participants’ focus was initially on their own needs and they described facing fear and having expectations of both developing and learning.

##### Facing fear

The participants described how starting the education meant leaving a safe haven and roles they were accustomed to as both lecturers and RNs. On the one hand, they felt that this made them more vulnerable in the new situation; on the other, they saw it as an opportunity to develop. This experience was likened to “being on an emotional roller-coaster”. They described feeling blindsided and shocked when they understood what the course was going to mean. This made them feel scared, in doubt, and insecure, but as one participant put it, “it was helpful to be really scared”.

*“It’s a mix of horror and delight in some way, yes absolutely it … I think I can do it, but can I get it together, with the rest of my life … I had not thought so much … before, what it would mean.” (FG 1)*

The feeling of insecurity was evoked by various parts of the course (e.g., how to arrange and pull together groups for supervision in clinical practice), but also by fears about failure, the future, and how to manage their everyday life and still have enough time for studies. The participants took on all the responsibility for all of this and experienced it as demanding. There was some scepticism about the online part of the course and worries about the technical equipment, internet connections, and the possibility of missing non-verbal communication.

##### Having expectations

The participants described an overall desire to learn, and starting the course came with different kind of expectations. One described it as “swinging out on the trapeze”. They conveyed the notion that being a supervisor in professional supervision in nursing was challenging and being a supervisor at all was a new function.

The participants described “having a personal agenda starting the course”, wanting to take on new challenges, move on, and develop.

*“I have some thoughts about … how this education might challenge me in new ways, in a role as a supervisor for nurses because I come from a psychiatric background, I have been involved in supervision in all sorts of variants … but I thought I … maybe that this could be something for me to take some steps further … // so this feels really exciting … ” (FG2)*

They had expectations about learning how to coach themselves, developing their communication skills, on finding solutions to personal dilemmas, and seeing new possibilities. They sought personal development and wanted to get to know their own strengths and weaknesses. They also expected to find balance within themselves. They described that in a way they felt chosen and said that taking the course was “an ego boost”, giving them a chance to advance and to receive supervision themselves. They also described it as an opportunity to slow down and decrease stress.

The participants who had earlier experiences of supervision had pronounced expectations of their development as supervisors. They expressed wanting to learn how to really listen and to take a step back, which they saw as a challenge in itself. They also wanted to develop preparedness, to acquire the tools for supervision, and learn how to hold fast. The participants also expressed expectations about helping others. They “wanted to learn how to support other people to be able to put words to their own solutions”, to reflect, and to believe in themselves.

To manage their expectations, the participants understood that the course was about learning by doing and they said that it felt safe to have opportunities to practice supervisory methods before starting their own groups. They saw the point of being challenged within a group of other students, getting help from each other, and taking part in each other’s knowledge. One participant said, “We are going to do this together”.

Overall, the participants appreciated giving of themselves and said that it felt interesting and exciting to meet challenging situations, both professional and personal. They had “expectations about being challenged” and expressed that “courage was required”. It felt challenging to take an online course, but the participants stated that it was about following time. They felt it was good that the course would take two years because they understood that their learning and development was to be “a process that was going to need time” for them to put their thoughts together.

#### Focusing on oneself in the group

The participants described having had ideals of their new role as supervisors and using the group as a means to reach their goal.

##### Having ideals of the role

The participants said that they had ideals about the role of supervisors and that the training gave them a model to develop their own platform in congruence with themselves and their ideals. They thought the role of supervisor was special and that it included being a role model for the group members. One said, “I got a new understanding of the role as supervisor”. The participants felt that they had supervision’gained new insight into how, as supervisors, they could influence their group members. They spoke about being in a position of power, where they would be able to control group members and have the power both to support them and, in a challenging way, to make them hurt them a little. They did not, however, want to harm anyone and “did not want strong emotions to scare group members away from sessions”. In relation to this, the participants expressed their need to be present and aware of when to take control and when to take a step back.

*“ I want tools so I can hold on to what happens and … maybe be able to handle it wisely, because there are times when I have left a conversation and felt completely unsuccessful, and thought to … So as a leader, this leadership quality, to hold on, can also be difficult, I want to train myself and get a little more preparedness for that.” (FG 2)*

The participants said that they used their new knowledge to develop their communication skills. They expected to be an example of how to formulate questions that foster reasoning. Furthermore, they were aware that different kinds of questions could yield different results and that they trained to ask questions without embedded interpretations and thought up counter questions. They trained to elicit further elaboration in conversations and to foster group members’ reflections. They were also aware of the importance of follow-up and continuing conversations by asking more questions.

The participants spoke about “conducting and achieving a basis of trust among the group members”. They wanted to invite members into a tolerant climate were they could feel safe and comfortable. This led the participants to think about responsibility and they described how the way they looked at responsibility had started to change. They saw it as their responsibility “to introduce and anchor shared responsibility to the group” and expected it to be challenging to get group members to understand this concept. The participants felt that a good supervisor should not draw too much attention during the sessions. Instead, a supervisor should shape the group to lead itself. The supervisor’s position should be one of listening, asking clarifying questions, and being present. Another key component mentioned was silence. The participants spoke about “daring to allow silence for reflection”. According to them, it required training to endure silence. The participants’ experiences were that silence was actually nice and that it felt like a strength to allow silence. They had also started to reflect on listening as a tool in supervision. They acknowledged that they really needed to listen to what was being said and to be ready with follow-up questions. Acting in that way was, according to the participants, more professional. They acknowledged that they needed to train themselves to stand back and to switch between being a listener and taking charge. The participants experienced that their training in listening affected how they acted even outside of supervision. Overall, the participants started to realize that, through their training, they were getting tools for both their professional and their private lives.

##### Using the group as a means

The participants spoke about how they used the group during their training. They stated that it was difficult to meet all the individuals in the group, but they also stated that they personally developed together in the group. Initially they felt afraid of meeting the group; they knew nothing of each other’s backgrounds, and it took courage to ask the group for help.

*“We are going to do this together in one way or another, and we are going to feel insecure and vulnerable … and I think that well we all are reasonable adults and I think we are going to support and help each other. Because it will be like you say, upside down, and sometimes you will be stressed by other things or feel that you are not prepared enough … and then it will be okay, I think, because we are just people … but that is development and we are going to do it together.” (FG 1)*

They also acknowledged that “how the group developed did not depend on one single person”. Instead, they noted that all group members shared responsibility for how the group developed. The participants expressed how shared responsibility felt like a relief and how as time went by they started to feel safe within the group.

Initially the participants used the course lecturer, who was also their own supervision group’s supervisor, as a role model for how to approach the person in focus. As they got more used to the model of professional supervision in nursing they started to call their own group “the safe group”. Still, they felt unsure of their abilities to supervise their own clinical supervision groups, and the thought of supervising own groups came with feelings of fear. The participants imagined their supervision within the course as mannered, different from clinical supervision in “reality”. They realized that they needed to strive for flow in their future groups and to get the group to understand that what they had together was important. For this to happen, the participants stated that several aspects such as the milieu were significant, but they were not convinced of their own ability to control those aspects.

Another quality of supervision that the participants raised was clarity. They spoke of “clarity as important regardless of having supervision in a room or online”. Structure needed to be embedded in the introduction and they recognized the need to have fixed groups over time and not too much time between sessions. The participants stated that “the group process was significant”, and they had not realized this before. Supervision required feeling safe with each other. The participants also wanted their future group members to leave feeling good and a with a sense of having received something. They wanted to see them develop and feel positive about themselves after supervision. The participants themselves described their own group as “the good group” and they hoped their future clinical group members would also take or have taken the course. They felt rewarded by receiving feedback and listening to others’ experiences of supervision, and they could already feel that it would be sad not to meet anymore.

#### Shifting perspectives on oneself and the group

The participants felt strengthened, appreciated travelling with the others on this journey, and experienced both their own group and the online room as safe places.

##### Feeling strengthened

The participants described the course as one of development. They stated that it contained both a personal journey and group belonging, and one described it as “an enriching multidimensional education that influenced me personally and professionally”. They even thought it affected their friends, like the lessons learnt spread and “became ripples on water”.

The participants described the course as fun and interesting. They stated that it gave them positive energy and counterbalanced their work situation. The course became “a lifeline” and they felt calmer and more trustful of themselves, more in control. They also described feeling safer, thankful, and somehow rested even though course had required a lot of work.

*”That is probably the delight, when it becomes obvious in the room … when I have dared to try new things … and it comes out just right, and that feeling … when I went home, yes it was like walking on clouds, and I lived on that feeling for a long time … that I dared and that the group was receptive … so it was a kick, a confidence, it was like a yes … I am comfortable in this, if I can fix this … then I can fix everything.” (FG 1)*

The participants described expressing themselves emotionally in academic writing as hard work, and at times, tiresome. Some said that they did not know what they had got themselves into taking the course. Being a student felt hard and challenging when they still had their ordinary work tasks to complete. Several of the participants did most of their studying after work on evenings and weekends, but it was time consuming and they did not get any for their studies. Some had expected the course to be something else, and some questioned whether the course really was something for them, as it was not just “a walk in the park”.

The participants expressed a feeling of having started to land. They felt grounded and noticed a change within themselves. They said that the lecturer of the course helped in this change and likened her to “a mother cat that sometimes bit us on the neck”. The participants talked repeatedly about their experiences of changing and being shaped. Sometimes they felt shaken, as changing one’s ways can chafe a bit. They spoke of the course as “brainwashing” and “reprogramming” them from their roles as nurses who give their patients advice and information. Nevertheless, they experienced this change to being more open and listening actively as something great.

The participants shared various experiences of the course. Some had felt prepared as they had heard about it being demanding and having several examinations. They expressed having had a lack of confidence in themselves, expecting it to be a bumpy road they were not sure would work out for them. However, they also said that the training had met their expectations and that it did not feel exhausting. They now had a better work situation with increased time for their own competence development. They liked writing, had time to finish the examinations, and therefore did not experience it as a lot of work.

The participants expressed having worried about stagnating, but also expectating personal development alongside professional development. They said that the training went beyond their expectations, however, as they now were better listeners and could ask questions in a more humble way. They recognized that they were more able to stay aware and calm, and found that the course had enriched them. They stated that they would carry this within themselves for the rest of their lives. The participants wanted to recommend the course to other nurses as they thought “every nurse should take it”. Furthermore, they thought the course should be part of overall nursing education as it would contribute to nursing students’ development as nurses.

##### Travelling with others

The participants felt it was an advantage to take this journey with the other students, and they wanted to continue meeting after the course. They were happy with the groups they had joined during the course and stated that everyone had been motivated. They also stated that the lecturer had a great impact on the group being “good”, and they that continuing meetings with the group were restful as the group felt safe. One said it was “fantastic” to be part of this milieu where people of different backgrounds met in a new arena. Listening to other people’s thoughts and reflections, learning from each other, was experienced as fruitful and valued as “the best part of the course”. They spoke about the group as “a room in itself” and about getting support from the group.

*”I am also very happy about the group … I have thought about this … everyone of us had a will and motivation I think, we have all been very motivated … it has been very restful to come to our meetings … incredibly restful … so in the sense that … yes you have come in then it is of course strenuous and by that I mean that you should like … empty yourself and be on your toes, learn and so on// … it have been very … we have been very safe … we have all been on that track.” (FG 1)*

##### Having a safe place

Both the group and “the room in cyber space” were described as safe places by the participants. They felt safe and close to the others regardless of where they actually were and tried to choose a calm place where they would not be disturbed. Joining the group while sitting at work, however, could be experienced as stressful.

*“I have not had the feeling that meeting each other in ’rooms’ online have mattered or played an important role. I have been very … close to the others even though we have sat in different ’rooms’. I have joined and they have been with me in my ’room’. It feels like I … have been very close and that we have had a very good exchange.” (FG 2)*

Although the participants generally met online to make this work, physical meetings were still expressed as a prerequisite. The participants emphasized the meaning of the once-a-semester on-campus meetings, stating that their relationships were deepened by these and that they would not want to be without them.

Starting the course came with worries about taking an online course. However, as time went by, the participants stopped reflecting on this. Instead they saw the possibilities of being able to gather and of more people being able to take the course as it did not require time-consuming travel or associated delays. One participant said, “there are more advantages than losses”. The participants found that they adapted to the situation and stopped thinking about where they sat during their meetings. They just noticed where the others sat and then “went into a bubble”. They were not disturbed by other things and tried to focus on the here and now, which required and gave them practice in focused listening.

The participants expressed how they were a bit sceptical in the beginning, worrying about missing out on non-verbal signals during their online conversations. However, what happened instead came as “a positive surprise”. The participants discovered that the methods they learned work very well online and they experienced fewer non-verbal disturbances than they had imagined. They described seeing each other’s faces and movements more clearer than they would have if in physical meetings. Sitting close to the computer also meant not being able to hide as they were constantly in each other’s focus. Furthermore, the participants felt they were able to know each other in a different way as they were able to “visit in their home environments” through the web cameras. Sometimes, “reality showed up” when something happened in the home when they were on camera together.

The participants described how meeting online could mean being less responsive as it was harder to sense the atmosphere of the room. There was “less small talk and the work started immediately when the meeting started”. The participants felt it was necessary for all to connect on time and to be present and disciplined. It was also necessary to have a stable internet connection and technical equipment. If such things did not work, it caused stress for the participants and disturbed the whole group.

The participants said that taking an online course was nothing unusual or odd. Taking the course online was not described as central, however, and it was not something they spontaneous thought about when they described the course to others. The participants did not experience the online parts of the course as disruptive; instead they spoke about them as being the future.

## Discussion

The participants’ experiences of learning to be a supervisor in professional supervision in nursing online was described as being on a personal and professional journey, echoing previous research on professional supervision (Borch et al., 2013; Francke & de Graaff, 2012). The participants in the present study described that in the beginning of the training they felt like novices with an overall desire to learn. Starting the course came with different kinds of expectations, and they expressed the notion that being a supervisor in professional supervision in nursing would be challenging and that being a supervisor was a new function to them. Overall, the participants expressed their appreciation of being able to give of themselves and their interest and excitement about daring to face challenging situations, both professional and personal. They expected to be challenged and that courage would be required. Initially it felt challenging to take an online course, but the participants stated it was the way of the future and therefore something to get used to.

Borsch et al. (2013) reported that structure and climate factors in the supervision model are considered key, partly in line with our results that showed the significans of the group and the teacher rather than the online environment in which the education was conducted. In the present study the participants described feeling socially and emotionally connected to the group. They experienced the course content as meaningful and reflected on the importance of both the group and the teacher as its structured facilitator. The online room and the group itself were experienced as safe places, and the social presence established within the group seems to have been significant to learning to be a YHIO supervisor. Garrison and Cleveland-Innes (2005) reported that interaction, communication, critical thinking, and reflection are seen as central to an educational experience and are often in focus in studies of online learning. Whether the education is conducted online, face-to-face, or in a blend of both, the purpose is to structure the educational experience to achive learning outcomes. Previous research highlighted the importance of creating a secure learning environment and facilitating reflection (Arvidsson & Fridlund, 2005). According to the CoI model (Garrison et al., 2000), online learning is assumed to happen through the interaction of social presence, cognitive presence, and teaching presence, which work together to support deep and meaningful inquiry and learning online (Garrison et al., 2001).

Martin et al. (2014) report that distance supervision can be perceived as a barrier to effective supervision, with a strong preference for face- to-face delivery. Our results show that the participants felt more vulnerable in a new situation that could also lead to opportunities for development. They had a clear self-focus and were shocked to discover what the course was to entail. This made them feel frightened, doubtful, and insecure However, they grew to appreciate being challenged within a safe group, helping each other, and sharing knowledge with each other. To support the benefits of learning online within a group, educators need to introduce and highlight the significance of social presence in online learning (Weidlich & Bastiaens, 2019). However, there are barriers and challenges to facilitating social presence, or the sense of “being there together” (Öztok & Kehrwald, 2017) in an online environment. Social presence requires mutual interdependence, a shared sense of belonging, connectedness, spirit, trust, interactivity, common expectations, shared values and goals, and overlapping histories among members (Rovai, 2002; Sung & Mayer, 2012). Trusting relationships are important in effective supervision and its subsequent benefits to the delivery of care. Integrity, respect, knowledge, confidence, congruity, and candour are essential to good supervision relationships (Arvidsson & Fridlund 2005). In the present study, the participants described the central components that fostered their learning process during the training. These components correspond to Martin et al. (2014) statement to use more than one mode of contact and Weidlich and Bastiaens (2019) description of designing sociable online learning environments and enhancing social presence. It includes providing online spaces and structured activities that encourage and support interaction as well as discussing social presence and establishing a commitment to interaction. The teacher needs to provide opportunities for individual presentations of thoughts, interactions between students, and group work. It is important to clarify the value of dialogue and collaborative learning. Group norms also need to be discussed and verified during the course (Weidlich & Bastiaens, 2019).

## Methodological discussion

The dependability of this study was strengthened by the selection of participants from students enrolled in the course Leadership and Professional Supervision in Nursing. The focus group discussions were conducted by the same person (BA), who is experienced in the field and encouraged the participants to talk openly. At the time for the mid focus group discussion, three participants were unable to participate, and at the last, four participants were absent. This may have affected the amount of data and therefore our opportunities to describe variations of experiences. However, the interviews were deemed rich and our reconstruction of data show variety of experiences among the participants.

Using data collected during 2014–2016 could mean not taking advantage of the latest developments. That is not the case in this study as the course runs part-time at 25% for four semesters and only has been completed twice since data collection. Based on our knowledge on the course, we have assessed data as current and therefore argue for its relevance. In addition, increased knowledge about online education is highly relevant as we now live in a time where such education is highly requested.

As a researcher, it is important to reflect on one’s own pre-understandings of the phenomenon in question. The authors examined their own pre-understandings through awareness-raising discussions and by reflecting on them when reading the interview texts. Three of the authors (JM, MÖ-N, B-ML) conducted the analysis and discussed the results until consensus was obtained. All authors participated in constructing the final findings. This process required genuine openness, flexibility, reflection, and critical discussions within the research group. The chosen quotations reflecting the content of each category offer the reader an opportunity to determine the confirmability of the study (Graneheim et al., 2017; Graneheim & Lundman, 2004). The study identifies and examines the phenomenon of how RNs experience learning to be a supervisor through studying professional supervision in nursing online.

## Conclusion and implications

The goal of nursing is to provide safe, high-quality care. To do that, nurses need to strengthen their professional role as nurses through supervision. Nurses today often work alone in complex environments with ambiguous responsibilities; therefore, access to supervision needs to be ensured. By our results we conclude that it is possible to learn how to become a supervisor via online tutoring. Implications are that online tutoring makes the course available for more RNs and this in turn can contribute to more RNs being offered supervision in the future. To pursue with online tutoring, it seems important to create social presence in this context. This can be done by providing online spaces, structured activities that encourage and support interaction, discussions about social presence, and an established commitment to interaction.
